# *Arsenophonus*, an emerging clade of intracellular symbionts with a broad host distribution

**DOI:** 10.1186/1471-2180-9-143

**Published:** 2009-07-20

**Authors:** Eva Nováková, Václav Hypša, Nancy A Moran

**Affiliations:** 1 Faculty of Science, University of South Bohemia, Branišovská 31, České Budějovice 37005, Czech Republic; 2 Faculty of Science, University of South Bohemia and Institute of Parasitology, Biology Centre of ASCR, Branišovská 31, České Budějovice 37005, Czech Republic; 3 Department of Ecology and Evolutionary Biology, The University of Arizona, 1041 E. Lowell St, Tucson, Arizona 85721-0088, USA

## Abstract

**Background:**

The genus *Arsenophonus *is a group of symbiotic, mainly insect-associated bacteria with rapidly increasing number of records. It is known from a broad spectrum of hosts and symbiotic relationships varying from parasitic son-killers to coevolving mutualists.

The present study extends the currently known diversity with 34 samples retrieved mainly from hippoboscid (Diptera: Hippoboscidae) and nycteribiid (Diptera: Nycteribiidae) hosts, and investigates phylogenetic relationships within the genus.

**Results:**

The analysis of 110 *Arsenophonus *sequences (incl. *Riesia *and *Phlomobacter*), provides a robust monophyletic clade, characterized by unique molecular synapomorphies. On the other hand, unstable inner topology indicates that complete understanding of *Arsenophonus *evolution cannot be achieved with 16S rDNA. Moreover, taxonomically restricted *Sampling *matrices prove sensitivity of the phylogenetic signal to sampling; in some cases, *Arsenophonus *monophyly is disrupted by other symbiotic bacteria. Two contrasting coevolutionary patterns occur throughout the tree: parallel host-symbiont evolution and the haphazard association of the symbionts with distant hosts. A further conspicuous feature of the topology is the occurrence of monophyletic symbiont lineages associated with monophyletic groups of hosts without a co-speciation pattern. We suggest that part of this incongruence could be caused by methodological artifacts, such as intragenomic variability.

**Conclusion:**

The sample of currently available molecular data presents the genus *Arsenophonus *as one of the richest and most widespread clusters of insect symbiotic bacteria. The analysis of its phylogenetic lineages indicates a complex evolution and apparent ecological versatility with switches between entirely different life styles. Due to these properties, the genus should play an important role in the studies of evolutionary trends in insect intracellular symbionts. However, under the current practice, relying exclusively on 16S rRNA sequences, the phylogenetic analyses are sensitive to various methodological artifacts that may even lead to description of new *Arsenophonus *lineages as independent genera (e.g. *Riesia *and *Phlomobacter*). The resolution of the evolutionary questions encountered within the *Arsenophonus *clade will thus require identification of new molecular markers suitable for the low-level phylogenetics.

## Background

**Figure 1:**
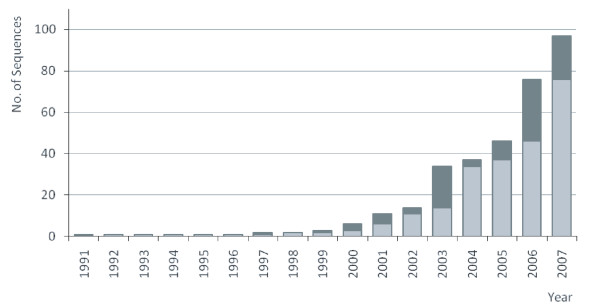
**An increase of records on *Arsenophonus *bacteria from various insect groups**. The bars show cumulative numbers of sequences deposited into GenBank; dark tops represent new records added in the given year. The sequences are identified with the following accession numbers: 1991 – M90801; 1997 – U91786; 2000 – AF263561, AF263562, AF286129, AB038366; 2001 – AF400474, AF400480, AF400481, AF400478, AY057392; 2002 – AY136168, AY136153, AY136142; 2003 – AY265341–AY265348, Y264663–AY264673, AY264677; 2004 – AY587141, AY587142, AY587140; 2005 – DQ068928, DQ314770–DQ314774, DQ314777, DQ314768, DQ115536; 2006 – DQ538372–DQ538379, DQ508171–DQ508186, DQ517447, DQ508193, DQ837612, DQ837613; 2007 – EU039464, EU043378, EF110573, EF110574, DQ076660, DQ076659, EF110572, EF647590, AB263104.

The bacterial genus *Arsenophonus *corresponds to a group of insect intracellular symbionts with a long history of investigation. Although many new *Arsenophonus *sequences have been published in the last several years, along with documentation of diverse evolutionary patterns in this group (Figure 1), the first records of these bacteria date to the pre-molecular era. Based on ultrastructural features, several authors described a transovarially transmitted infection associated with son-killing in the parasitoid wasp *Nasonia vitripennis *[1-3]. Later, they were formally assigned to a new genus within the family Enterobacteriaceae with a single species, *Arsenophonus nasoniae *[4]. The same authors proposed a close relationship of *Arsenophonus *to free-living bacteria of the genus *Proteus*. Independently, other microscopic studies revealed morphologically similar symbionts from various tissues of blood-sucking triatomine bugs [5,6]; a decade later these bacteria were determined on molecular grounds to belong to the same clade and were named *Arsenophonus triatominarum *[7]. Interestingly, the next record on symbiotic bacteria closely related to *A. nasoniae *was from a phytopathological study investigating marginal chlorosis of strawberry [8]. Since available sequence data were insufficient for reliable phylogenetic placement, the phloem-inhabiting pathogen was described as a new genus, *Phlomobacter*, with a single species *P. fragariae *[8].

Since these descriptions, the number of *Arsenophonus *records has steadily been increasing, resulting in two important changes in knowledge of *Arsenophonus *evolution and roles in hosts. First, the known host spectrum has been considerably extended with diverse insect groups and even non-insect taxa. So far, *Arsenophonus *has been identified from parasitic wasps, triatomine bugs, psyllids, whiteflies, aphids, ticks, ant lions, hippoboscids, streblids, bees, lice, and two plant species [4,7-23]. Second, these recent studies have revealed an unsuspected diversity of symbiotic types within the genus. This dramatically changes the original perception of *Arsenophonus *as a bionomically homogeneous group of typical secondary ("S-") symbionts undergoing frequent horizontal transfers among phylogenetically distant hosts. For example, recent findings indicate that some insect groups harbor monophyletic clusters of *Arsenophonus*, possibly playing a role of typical primary ("P-") symbionts. These groups were reported from the dipteran families Hippoboscidae and Streblidae [20] and most recently from several lice species [18,24,25]. Such a close phylogenetic relationship of different types of symbiotic bacteria is not entirely unique among insect symbionts. With the increasing amount of knowledge on the heterogeneity and evolutionary dynamics of symbiotic associations, it is becoming clear that no distinct boundaries separate the P- and S-symbionts. Thus, in their strict meaning, the terms have recently become insufficient, especially for more complex situations, such as studies exploring bacterial diversity within a single host species [14,17]. Furthermore, these terms have been shown not to reflect phylogenetic position; remarkable versatility of symbiotic associations can be observed in the Gammaproteobacteria overall, as well as within the individual clusters, such as *Arsenophonus *or *Sodalis *[16,26].

The genus *Arsenophonus *is striking in the diversity of symbiont types represented. Apart from many lineages with typical S-symbiont features, this genus has given rise to several clusters of P-symbionts [18,20,24]. Unfortunately, this heterogeneity introduced an annoying degree of phylogenetic instability and nomenclatory confusion. Because P-symbionts show accelerated evolutionary rates, they form long branches in phylogenies, leading to unstable patterns of clustering as observed for P-symbionts within Enterobacteriaceae [27]. The same behavior can be seen in the louse-specific clade of *Arsenophonus*, which are consequently originally described as a new bacterial genus *Riesia *[25]. In addition, the *Arsenophonus *cluster is the only monophyletic group of symbiotic bacteria currently known to possess at least four highly different phenotypes, including son-killing [4], phytopathogenicity [8], obligate association with bacteriocytes in the host [18,20,24], and apparently non-specific horizontally transmitted bacteria that are possibly mutualistic [15]. These characteristics indicate that the genus *Arsenophonus *represents an important and widespread lineage of symbiotic bacteria that serves as a valuable model for examining molecular evolution of bacteria-arthropod associations.

In this study, we add 34 new records on symbionts to the known spectrum of *Arsenophonus *lineages. We explore and summarize the current picture of *Arsenophonus *evolution by analyzing all sequences available for this clade. To investigate the phylogenetic position, stability and evolutionary trends of the *Arsenophonus *cluster, we complete the sample with related symbionts and free-living bacteria. Finally, we explore molecular characteristics and informative value of the 16S rRNA gene as the most frequently used phylogenetic marker.

## Results

### Sequences and alignments

**Figure 2:**
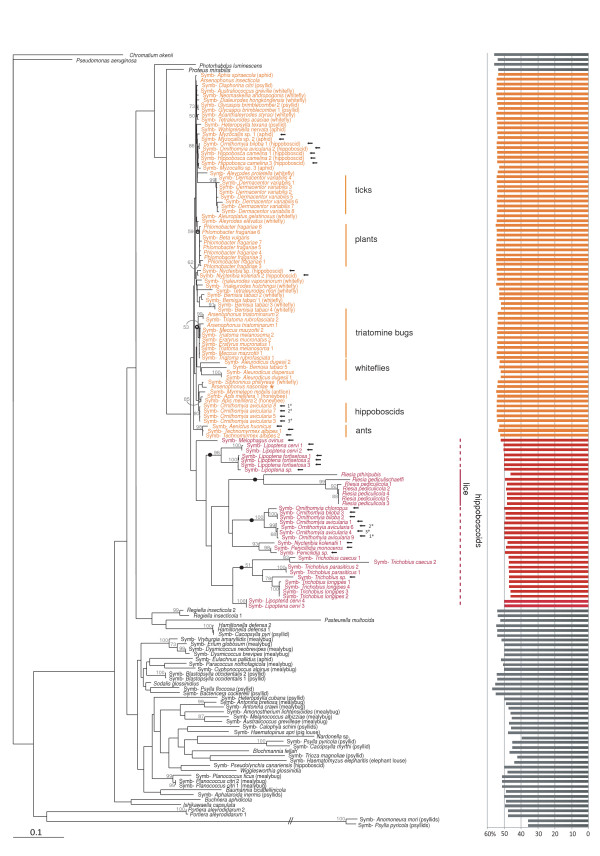
**Phylogenetic tree derived from the *Basic matrix *(1222 positions) under ML criterion**. Names of the taxa clustering within the *Arsenophonus *clade are printed in colour: red for the long-branched taxa, dark orange for the short-branched taxa. The arrows point to the new sequences obtained in our study. Different types of sequences determined from the specimens of *O. avicularia *are designated by the numbers with asterisks. The type species *A. nasoniae *is designated by the orange asterisk. Solid circles on branches label the clusters strictly concordant with the host phylogenies. Open circles designate host-specific lineages without coevolutionary signal. Solid vertical lines indicate reciprocally monophyletic groups of symbionts and hosts. Dashed lines show paraphyletic symbiont clades restricted to monophyletic host groups. Names in the brackets indicate host taxa. "Symb-" in the taxon designation stands for "Symbiotic bacteria of". Bars represent GC content of each taxa. Complete information on the sequences is provided in the Additional file 5.

From 15 insect taxa, we obtained 34 sequences of 16S rDNA that exhibited a high degree of similarity to sequences from the bacterial genus *Arsenophonus *when identified by BLAST. The length of the PCR-amplified fragments varied from 632 to 1198 bp, with the guanine-cytosine (GC) content ranging from 46.22 to 54.84% (Figure 2, bars). For three specimens of the hippoboscid *Ornithomya avicularia*, two different sequences were obtained from each single individual. After combining with all *Arsenophonus *16S rDNA sequences currently available in the GenBank, and several additional free-living and symbiotic bacteria, the dataset produced a 1222 bp long *Basic matrix*. The alignment has a mosaic structure, discussed below. Within the set, a large group of sequences show a high degree of similarity (0.1–7.3% divergence) and exhibit GC content and sequence length similar to those found in free-living enterobacteria. The set also includes several sequences with modifications typical for many proteobacterial symbionts, particularly the presence of long insertions within the variable regions and decreased GC content. Sequence distances among these taxa range up to 17.8%.

### Phylogeny

All phylogenetic analyses of the *Basic matrix *yielded a monophyletic *Arsenophonus *clade (Figure 2). The new 34 sequences (Figure 2, arrows), identified by BLAST as putative members or relatives of the genus *Arsenophonus*, always clustered within the *Arsenophonus *clade. Their precise position was only partially correlated with host taxon. Some of the *Arsenophonus *sequences from hippoboscoid hosts clustered within monophyletic host-specific groups (Figure 2, printed in red) while others were scattered across the tree as isolated lineages (Figure 2, printed in dark orange). Two distinct sequences were determined from each individual specimen of *O. avicularia*; these clustered at distant positions within the tree (Figure 2, numbers with asterisks).

The most typical lineages display short-branches with low divergence and unstable positions within the *Arsenophonus *clade (Figure 2, printed in dark orange). At the opposite extreme are well supported host-specific clusters exhibiting long branches, such as the louse symbiont *Riesia *or the symbionts described from several streblid species. An intermediate situation is found in putatively host-specific but less robust clusters, such as the *Arsenophonus *lineages from triatomine bugs, some hippoboscoids or homopterans (Figure 2). In an analogy to previously analyzed symbiotic bacteria [e.g. [28,29]], the phylogenetic properties of the sequences were also reflected in their GC contents. In the short-branched taxa, the GC content of the 16S rRNA sequence varies from 51.72 to 54.84%, the values typical for S-symbionts and free-living bacteria [30]. In contrast, the 16S rRNA sequences with low GC content, varying between 46.22 and 51.93%, were found in the long-branched taxa clustering within the host-specific monophyletic lineages (e.g. the symbionts from *Ornithomyia*, *Lipopten*a, *Trichobius*, and the *Riesia *clade).

Considerable loss of phylogenetic information was observed in the *Conservative matrix*. In this case, the relationships among individual *Arsenophonus *lineages were highly unstable, resulting in large polytomies of many short-branched taxa within the consensus trees (see Additional file 1). Also the relationships among the long-branched lineages, although resolved, differ sharply from those derived from the *Basic matrix *data, and the genus *Proteus *was not positioned as the closest relative of *Arsenophonus*. Thus, the information contained in the *Conservative matrix *(restricted to one fourth of *Basic *dataset, i.e., 284 bp) is insufficient for reliable phylogenetic placement of closely related taxa.

**Figure 3:**
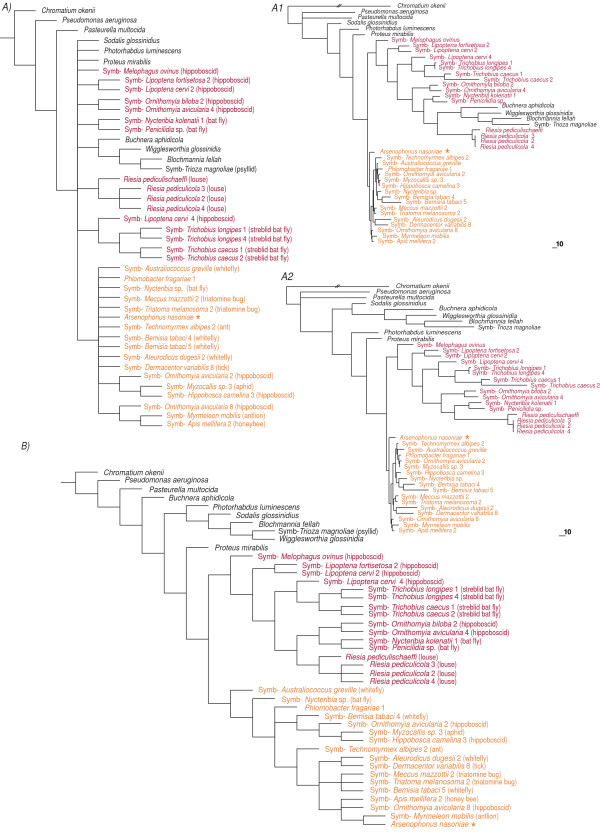
**Topologies derived from *Sampling3 *matrix (851 positions)**. A) consensus of the trees and two tree examples A1 and A2, obtained under the MP criterion with Tv/Ts ratio set to 1:1 B) consensus of the trees obtained under the MP criterion with Tv/Ts ratio set to 1:3. The type species *A. nasoniae *is designated by the orange asterisk.

**Figure 4:**
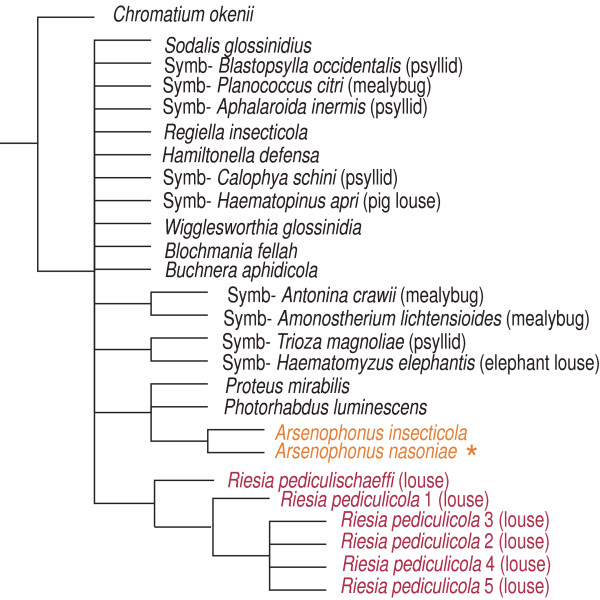
**Tree consensus derived from *Sampling5 *(936 positions) matrix under the MP criterion**. Transversion/transition ratio was set to 1:1. The type species *A. nasoniae *is designated by the orange asterisk.

The analyses of taxonomically restricted *Sampling *matrices confirmed the expected dependence of the phylogenetic conclusions on the taxon sampling (examples of topologies obtained are provided in Figures 3, 4 and Additional file 2). The highest degree of susceptibility was observed with MP, particularly under Tv:Ts ratio set to 1. The most fundamental distortion occurred with the matrix *Sampling3*, where one lineage (composed of *Buchnera*, *Wigglesworthia*, *Blochmannia*, and S-symbiont from *Trioza magnoliae*) clustered either as a sister group of *Riesia *clade or together with *Sodalis*. Thus, the consensus tree did not preserve the monophyly of an *Arsenophonus *clade (Figure 3).

The calculation of divergence times yielded substantially different results depending on the choice of calibration points. Use of the *Riesia *diversification as a reference point suggested a recent origin of the triatomine-associated *Arsenophonus *branch; the median value of the estimate distribution was 2.6 mya. In contrast, the calibration by *Escherichia-Salmonella *returned considerably higher dates with the median at 24.5 mya.

## Discussion

### Phylogenetic patterns and the stability of the information

**Figure 5:**
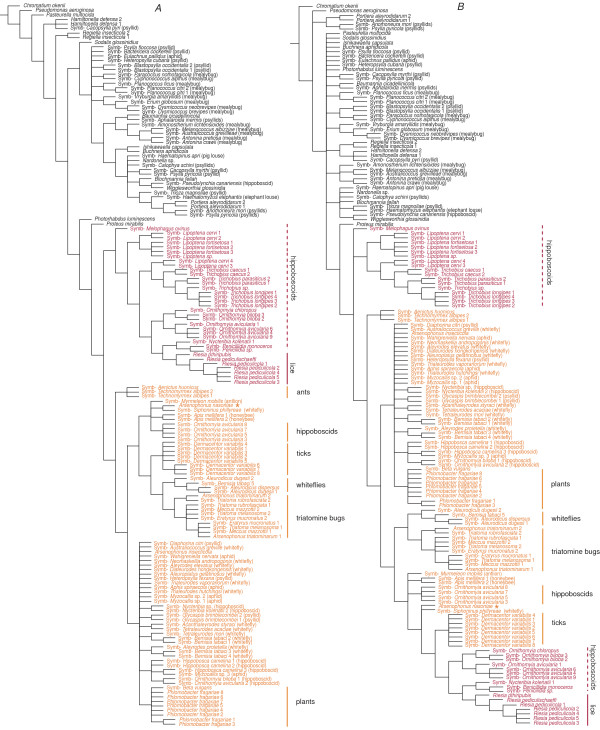
**Topologies derived from the *Basic matrix *(1222 positions)**. A) consensus of the trees obtained under the MP criterion with transversion/transition ratio set to 1:3 and the ML criterion; B) consensus of the MP trees obtained with the transversion/transition ratio 1:1. The type species *A. nasoniae *is designated by the orange asterisk.

**Figure 6:**
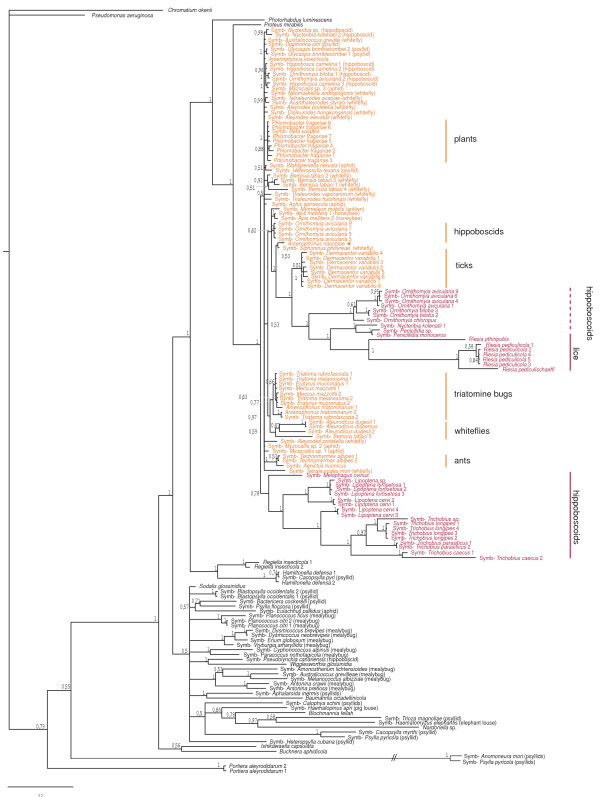
**Phylogenetic tree derived from *Basic matrix *(1222 positions) using Bayesian analysis**. Names of the taxa clustering within the *Arsenophonus *clade are printed in colour: red for the long-branched taxa, dark orange for the short-branched taxa. Names in the brackets designate the host family. Numbers represent Bayesian posterior probability for each node. The type species *A. nasoniae *is designated by the orange asterisk.

Phylogenetic relationships of the *Arsenophonus *symbionts display a remarkably complex arrangement of various types of symbiosis and evolutionary patterns. Moreover, a comparison of the branch ordering within each of these subclusters to the host phylogeny indicates a cospeciation process within several lineages (discussed below). From the phylogenetic perspective, no clearcut boundary divides the set of *Arsenophonus *sequences into the ecologically distinct types. The position of the long-branched subclusters within the topology is not stable. Under the MP criterion with transition rate 1:3 and under the ML criterion they form a unique monophyletic cluster (Figure 5A), while in other analyses the individual host-specific subclusters were scattered among the short-branched lineages (Figure 5B, Figure 6).

The low resolution and instability of the trees inferred from the *Conservative matrix *suggest that a substantial part of the phylogenetic information is located within the "ambiguously" aligned regions that were removed by the GBlocks procedure. This fact is particularly important when considering the frequent occurrence of insertions/deletions within the sequences (see Additional file 3). This may lead to deletion of these critical fragments in many phylogenetic analyses. Interestingly, the monophyletic nature of *Arsenophonus *was preserved even in this highly *Conservative matrix*. This indicates that within the complete data set, the phylogenetic information underlying the *Arsenophonus *monophyly is sufficiently strong and is contained in the conservative regions of the sequences. In accordance with this presumption, several molecular synapomorphies can be identified in the *Basic *and *Conservative *matrices. The most pronounced is the motif GTC/GTT located in positions 481–483 and 159–161 of *Basic matrix *and *Conservative matrix*, respectively.

### Relevance of the sampling

To test an effect of sampling on the phylogenetic inference within *Arsenophonus*, we examined five *Sampling *matrices with different taxa compositions (see the section *Methods*). In addition to the MP, ML, and Bayesian analyses, we performed an ML calculation under the nonhomogeneous model of the substitutions, designated as T92 [31,32]. This model was previously used to test the monophyly/polyphyly of the P-symbiotic lineages and brought the first serious evidence for a possible independent origin of major P-symbiotic taxa [27]. We were not able to apply the same approach to the *Basic *and *Conservative *matrices since the program Phylowin failed to process these large datasets under the ML criterion. The analyses of several taxonomically restricted *Sampling *matrices proved the sensitivity of phylogenetic signal to the sampling. In the most extreme case, shown in Figure 3A, even the monophyly of the *Arsenophonus *clade was disrupted by other lineages of symbiotic bacteria. Considering the results of the extensive analysis of the *Basic matrix*, this arrangement is clearly a methodological artifact. Since both *Riesia *and the P-symbiont lineage are long-branched taxa with rapid evolution of 16S rDNA, their affinity is very likely caused by Long Branch Attraction (LBA; for review see [33]) within the taxonomically compromised matrix. It is symptomatic that this topology was inferred by MP, the method known to be particularly prone to the LBA. To further test this distortion, one of the long-branched taxa was removed from the data set (matrix *Sampling4*). This approach restored the *Arsenophonus *monophyly and confirmed the effect of LBA phenomenon (see Additional file 2).

The aim of these taxonomically restricted analyses was to "simulate" phylogenetic placement of newly determined symbionts. In such casual studies, the symbiotic lineages are rarely represented by all available sequences in the way we composed the *Basic matrix*. Rather, each symbiotic lineage is represented by few randomly selected sequences. Under such circumstances, incorrect topologies (e.g. the *Sampling5*-derived topology on the Figure 4) can be obtained due to various methodological artifacts. This situation can be illustrated by empirical data: at least in two studies, the louse-associated lineage of *Arsenophonus *was not recognized as a member of the *Arsenophonus *clade [25,34]. Consequently, when more recent studies, based on better sampling, proved the position of *Riesia *within the *Arsenophonus *cluster [18,24] the genus *Arsenophonus *became paraphyletic (see the section *Conclusion *for more details).

Interestingly, topologies inferred by likelihood analyses using the T92 evolution model [31] were influenced neither by the compromised sampling nor by the removal of unreliably aligned regions.

### Cophylogeny vs. horizontal transfers: possible sources of phylogenetic incongruence

The phylogenetic tree of all *Arsenophonus *sequences exhibits both patterns, the parallel evolution of symbionts and their hosts and the haphazard association of symbionts from different host taxa. Coincidentally, both arrangements can be demonstrated on the newly sequenced symbionts from various hippoboscoid species. Some of hippoboscoid-associated *Arsenophonus *show possible host specificity; in a few analyses they cluster within several monophyletic short-branched groups. Since relationships among the short-branched taxa are generally not well resolved, these lineages are scattered throughout the whole topology (Figure 2). In contrast, relationships within the long-branched clusters of hippoboscoid-associated taxa are in agreement with the host phylogeny (the *Arsenophonus *clusters strictly reflecting the host phylogeny are designated by solid circle in the Figure 2). Interestingly, a coevolutionary pattern was also identified for streblids of the genus *Trichobius *and their symbionts. In the original study published by Trowbridge et al. [20], the distribution of *Trichobius *symbionts was apparently not consistent with the host phylogeny. Our analysis in a broad context indicates that this discrepancy might have been caused by different bacterial sampling and particularly by aberrant behavior of the sequence from *Trichobius yunkeri *[GenBank: DQ314776]. This sequence is likely to be an artificial chimerical product of at least two distant lineages; according to our BLAST tests it shares 100% identity with S-symbiont of *Psylla pyricola *[GenBank: AF286125] along a 1119 bp long region. Removal of this sequence from the dataset restored a complete phylogenetic congruence between *Trichobius*, based on the phylogeny of this genus published by Dittmar et al. [35], and its symbionts. This finding exemplifies the danger of chimeric sequences in studies of symbiotic bacteria, obtained by the PCR on the sample containing DNA mixture from several bacteria. The presence of several symbiotic lineages within a single host is well known [e.g. [14,36-38]]. In this study, we demonstrate a possible such case in *O. avicularia*. From three individuals of this species we obtained pairs of different sequences branching at two distant positions (labelled by the numbers 1* to 3* in Figure 2). The identical clustering seen in all three pairs within the tree shows that they are not chimeric products but represent two different sequences.

While the identity between symbiont relationships and the host phylogeny is apparently a consequence of host-symbiont cophylogeny, the interpretation of the randomly scattered symbionts is less obvious. Usually, such an arrangement is explained as result of transient infections and frequent horizontal transfers among distant host taxa. This is typical, for example, of the *Wolbachia *symbionts in wide range of insect species [39]. Generally, the capability to undergo inter-host transfers is assumed for several symbiotic lineages and has even been demonstrated under experimental conditions [40,41]. Since the *Arsenophonus *cluster contains bacteria from phylogenetically distant insect taxa and also bacteria isolated from plants, it is clear that horizontal transfers and/or multiple establishments of the symbiosis have occurred. However, part of the incongruence could be caused by methodological artifacts. A conspicuous feature of the *Arsenophonus *topology is the occurrence of monophyletic symbiont lineages associated with monophyletic groups of insect host but without a co-speciation pattern. Although our study cannot present an exhaustive explanation of such a picture, we want to point out two factors that might in theory take part in shaping the relationships among *Arsenophonus *sequences, lateral gene transfer (LGT) and intragenomic heterogeneity. Both have previously been determined as causes of phylogenetic distortions and should be considered in coevolutionary studies at a low phylogenetic level.

### Incongruence due to LGT and intragenomic heterogeneity

An apparently "mosaic" structure of the *Arsenophonus *alignment (for example see Additional file 4) raises the question of whether various regions of this sequence could have undergone different evolutionary histories. Recombination of 16S rDNA genes were previously identified in some other bacteria [42-44]. In actinomycetes, the occurrence of short rDNA segments with high number of non-random variations was attributed to the lateral transfer as the most parsimonious explanation [45]. Later, Gogarten et al. [46] suggested that, analogously to an entire bacterial genome, 16S rDNA possesses a mosaic character originated by LGT, respectively by transfer of gene subunits.

As bacterial genomes often carry more than one rRNA operon, intragenomic heterogeneity of the rDNA copies is occasionally found to blur the phylogenetic picture [47-50]. Although there is no direct information on the number of rRNA gene copies in *Arsenophonus *genomes, Stewart and Cavanaugh [51] showed bacterial genomes to encode in average five rRNA operons. The most closely related bacterium of which the complete genome has recently been sequenced, *Proteus mirabilis*, carries seven copies [GenBank: AM942759]. *Arsenophonus*-focused studies indicate that two different forms of the rRNA operon are present in its genome, as is typical for Enterobacteraceae [23,52]. Furthermore, Šorfová et al. [23] suggest that the variability among individual copies may cause the incongruence observed between triatomines and their *Arsenophonus *lineages. They point out that this process could, in principle, explain an otherwise problematic observation: in some hosts, such as triatomines or some homopterans, the hosts and the *Arsenophonus *bacteria create reciprocally monophyletic clusters but do not show any cospeciation pattern. In the symbionts of grain weevils, divergence between rRNA sequences within a genome was shown sometimes to exceed divergence of orthologous copies from symbionts from different hosts; this unusual situation was hypothesized to reflect loss of recombinational repair mechanisms from these symbiont genomes [53].

### Estimates of the divergence time

With the present incomplete knowledge of the *Arsenophonus *genome, it is difficult to assess whether and how deeply rRNA heterogeneity affects phylogenetic reconstruction. Trying to find alternative solution, Šorfová et al. [23] attempted to use the estimation of divergence times as a guide for deciding between different coevolutionary scenarios. They used the *Escherichia-Salmonella *divergence [54,55] as a calibration point for calculating the divergence time among various *Arsenophonus *lineages from triatomine bugs. Applying the Multdiv method [56], they placed the ancestor of triatomine-associated symbionts into a broad range of approx. 15 – 40 mya and concluded that this estimate is compatible or even exceeds the age estimates available for the tribe triatomine (according to Gaunt and Miles [57]). Here, we took advantage of a new age-estimate for closely related bacteria, namely the louse-associated symbionts of the genus *Riesia *[18]. Comparing the estimates based on the two calibration methods (*Escherichia-Salmonella *and *Riesia*), we found that due to the variability of evolutionary rates among the lineages, the results may differ by an order of magnitude. Such marked variance among different bacterial lineages (including different symbiotic bacteria from the same host species) was previously reported for many bacterial groups [29,30,37,39,58-63]. Most recently, Allen et al. [64] reported an extremely high evolutionary rate for the young symbiotic lineage *Riesia*, and suggested that the evolutionary tempo changes with the age of the symbiotic lineage. We therefore conclude that this method cannot be directly used to assess the effect of intragenomic heterogeneity on our reconstruction of *Arsenophonus *relationships.

## Conclusion

With more than one hundred records, the genus *Arsenophonus *represents one of the richest and most widespread clusters of insect symbiotic bacteria. Considering its broad host spectrum and apparent ecological versatility, *Arsenophonus *should play an important role in studies of evolutionary trends in insect intracellular symbionts. Due to this fact, *Arsenophonus *is likely to attract a growing attention, and the number of the records may rapidly be increasing during the next years. For example, 7 new sequences were deposited into the GenBank since the completion of this study [[65], and unpublished record FJ388523]. However, since these new *Arsenophonus *records originated in screening rather than phylogenetic study, they are only represented by short DNA fragments (approx. 500 bp). Preliminary analyses of these fragments together with our complete datasets confirmed a limited informative value of such short sequences and they were not included into the more exhaustive phylogenetic procedures.

The analysis of 110 available sequences of *Arsenophonus *16S rDNA from 54 host taxa revealed several interesting evolutionary patterns. In particular, this clade includes at least two transitions from S-symbiont, with ability to invade new host lineages, to P-symbiont, showing obligate relationship to hosts and a strict pattern of maternal transmission. Thus, it is a promising system for exploring the genomic and biological changes that accompany the shift from facultative to obligate symbiont. *Arsenophonus *is also one of the few groups of insect symbionts for which strains have been grown in pure culture [4,7,16], a feature that further enhances its potential as a model for symbiont research.

Our results also indicate that a complete understanding of the *Arsenophonus *phylogeny cannot be achieved with 16S rDNA genes alone. A similar situation is, for example, found in another large symbiotic group, the genus *Wolbachia*, where other genes are often used as alternative sources of phylogenetic information [66,67]. Identification of suitable low-level-phylogeny marker(s) is thus one of the most crucial steps in the further research on *Arsenophonus *evolution. The sequencing of the complete *Arsenophonus *genome, which is currently under the process http://genomesonline.org/gold.cgi?want=Bacterial+Ongoing+Genomes&pubsort=Domain, will provide a valuable background for such enterprise.

Based on the presented analyses, we also want to point out that the genus *Arsenophonus *is currently paraphyletic due to the two lineages described as separate genera *Riesia *and *Phlomobacter *but clustering within the *Arsenophonus *group (e.g. Figure 2). Two procedures can, in principle, solve this undesirable situation, splitting of the *Arsenophonus *cluster into several separate genera or classification of all its members within the genus *Arsenophonus*. Taking into account the phylogenetic arrangement of the individual lineages, the first approach would inevitably lead to establishment of many genera with low sequence divergences and very similar biology. The second option has been previously mentioned in respect to the genus *Phlomobacter *[68], and we consider this approach (i.e. reclassification of all members of the *Arsenophonus *clade within a single genus) a more appropriate solution of the current situation within the *Arsenophonus *clade.

## Methods

### Samples

The host species used in this study were acquired from several sources. All of the nycteribiid samples were obtained from Radek Lučan. Most of the hippoboscids were provided by Jan Votýpka. Ant species were collected by Milan Janda in Papua New Guinea. All other samples are from the authors' collection. List of the sequences included in the *Basic matrix *is provided in the Additional file 5.

### DNA extraction, PCR and sequencing

The total genomic DNA was extracted from individual samples using DNEasy Tissue Kit (QIAGEN; Hilden, Germany). Primers F40 and R1060 designed to amplify approx. 1020 bp of 16S rDNA, particularly within Enterobacteriaceae [34], were used for all samples. PCR was performed under standard conditions using HotStart Taq polymerase (HotStarTaqi DNA Polymerase, Qiagen). The PCR products were analyzed by gel electrophoresis and cloned into pGEM-T Easy System 1 vector (Promega). Inserts from selected colonies were amplified using T7 and SP6 primers and sequenced in both directions, with the exception of 3 fragments sequenced in one direction only (sequences from *Aenictus huonicus *and *Myzocalis sp*.). DNA sequencing was performed on automated sequencer model 310 ABI PRISM (PE-Biosystems, Foster City, California, USA) using the BigDye DNA sequencing kit (PE-Biosystems). For each sample, five to ten colonies were screened on average. The contig construction and sequence editing was done in the SeqMan program from the DNASTAR platform (Dnastar, Inc. 1999). Identification of the sequences was done using BLAST, NCBI http://www.ncbi.nlm.nih.gov/blast/Blast.cgi.

### Alignments

To analyze thoroughly the behavior of *Arsenophonus *16S rDNA and assess its usefulness as a phylogenetic marker, we prepared several matrices and performed an array of phylogenetic analyses on each of them.

The *Basic matrix *was composed of the 34 new sequences, all *Arsenophonus *sequences available in the GenBank and additional 45 sequences of various P-symbionts, S-symbiont and 5 free living bacteria (see Additional file 5). To show the impact of random or restricted sampling on the resulting topology, five different matrices labelled *Sampling*_*i *_(i.e. *Sampling1*, *Sampling2*, etc.) were prepared from *Basic matrix *by removing various taxa and including additional/alternative outgroups. The matrices *Sampling1 *to *Sampling4 *were composed of various numbers of non-*Arsenophonus *symbiotic taxa (ranging from 3 to 35), three sequences of free-living bacteria, and an arbitrarily selected set of all *Arsenophonus *lineages. Matrix designated as *Sampling5 *was restricted to a lower number of taxa, including 5 ingroup sequences and alternative lineages of symbiotic and free-living bacteria.

All matrices were aligned in the server-based program MAFFT http://align.bmr.kyushu-u.ac.jp/mafft/online/server/, using the E-INS-i algorithm with default parameters. The program BioEdit [69] was used to manually correct the resulting matrices and to calculate the GC content of the sequences.

To test an effect of unreliably aligned regions on the phylogenetic analysis, we further prepared the *Conservative matrix*, by removing variable regions from the *Basic matrix*. For this procedure, we used the program Gblocks [70] available as server-based application on the web page http://molevol.cmima.csic.es/castresana/Gblocks_server.html.

Finally, the *Clock matrix*, composed of 12 bacterial sequence (see Additional file 5), was designed to calculate time of divergence for several nodes within the *Arsenophonus *topology.

### Phylogenetic analyses

The matrices were analyzed using maximum parsimony (MP), maximum likelihood (ML) and Bayesian probability. For analyses, we used the following programs and procedures. *The GTR+Γ+inv *model of molecular evolution was determined as best fitting by the program Modeltest [71] and was used in all ML-based analyses. MP analysis was carried out in TNT program [72] using the *Traditional search *option, with 100 replicates of heuristic search, under the assumptions of Ts/Tv ratio 1 and 3. ML analysis was done in the Phyml program [73] with model parameters estimated from the data. Bayesian analysis was performed in Mr. Bayes ver. 3.1.2. with following parameter settings: nst = 6, rates = invgamma, ngen = 3000000, samplefreq = 100, and printfreq = 100. The program Phylowin [74] was employed for the ML analysis under the nonhomogeneous model of substitution [31].

A calculation of divergence time was performed in the program Beast [75] which implements MCMC procedure to sample target distribution of the posterior probabilities. The gamma distribution coupled with the *GTR+invgamma *model was approximated by 6 categories of substitution rates. Relaxed molecular clock (uncorrelated lognormal option) was applied to model the rates along the lineages. To obtain a time-framework for the tree, we used the estimate on louse divergence (approximately 5.6 mya [18]). Since the resulting estimate was considerably lower that that reported previously with *Escherichia-Salmonella *calibration [23], we prepared an additional matrix and used the *Escherichia-Salmonella *split [54,55] as an alternative calibration; taxa included according to Šorfová et al. [23]. All analyses were performed in three independent runs, each taking 5 million generations.

## Authors' contributions

EN obtained the sequence data, compiled alignments and participated in the study design, phylogenetic inference, interpretation of the results, and preparation of the manuscript. VH conceived of the study and participated in conduction of the phylogenetic inference. Both, VH and NAM participated in the study design, evolutionary interpretation of the results and preparation of the manuscript. All authors read and approved the final manuscript.

## Supplementary Material

Additional file 1 **Consensus tree derived from the *Conservative matrix *(284 positions) under MP criterion**. Transversion/transition ratio was set to 1:3. Names of the taxa clustering within the *Arsenophonus *clade derived from *Basic matrix *are printed in colour: red for the long-branched taxa, dark orange for the short-branched taxa. Click here for file

Additional file 2 **Tree consensus derived from *Sampling4 *(1107 positions) matrix under the MP criterion**. Transversion/transition ratio was set to 1:1. The type species *A. nasoniae *is designated by the orange asterisk. Click here for file

Additional file 3 Insertions within the sequences of P-like symbionts. Click here for file

Additional file 4 **The 41 bp long motif inconsistently distributed among distinct bacterial taxa**. Position of the sequence in alignment and 16S rDNA secondary structure is indicated by the arrows. Following records are not included in the Additional file 1: *Sitophilus rugicollis *[GenBank: AY126639], *Drosophila paulistorum *[GenBank: U20279, U20278], *Polyrhachis foreli *[GenBank: AY336986], *Haematopinus eurysternus *[GenBank: DQ076661]. Click here for file

Additional file 5 **List of sequences included in *Basic matrix***. Dashed line separates members of the *Arsenophonus *clade from the outgroup taxa. Sequences included into the *Clock matrix *are underlined. Click here for file
