# Supplementary material for: Arsenophonus, an emerging clade of intracellular symbionts with a broad host distribution
Source: BMC Microbiol. 2009 Jul 20;9:143. doi: 10.1186/1471-2180-9-143 (PMC2724383; doi:10.1186/1471-2180-9-143)
Supplement: Additional file 5 — List of sequences included in Basic matrix. Dashed line separates members of the Arsenophonus clade from the outgroup taxa. Sequences included into the Clock matrix are underlined. [file 1471-2180-9-143-S5.doc]

| **Host Species** | **Symbiont designation** | **Acc. No.** |  | **Host Species** | **Symbiont designation** | **Acc. No.** |  | **Host Species** | **Symbiont designation** | **Acc. No.** |
| --- | --- | --- | --- | --- | --- | --- | --- | --- | --- | --- |
|  |  |  |  |  |  |  |  |  |  |  |
| *Hippobosca camelina* | *Hippobosca camelina* 1 | FJ265793 |  | *Trichobius caecus* | *Trichobius caecus 2* | DQ314768 |  | *Eratyrus mucronatus* | *Eratyrus mucronatus* 1 | DQ508198 |
|  | *Hippobosca camelina* 2 | FJ265792 |  | *Australiococcus greville* | *Australiococcus greville* | AY264673 |  | *Eratyrus mucronatus* | *Eratyrus mucronatus* 2 | DQ508182 |
|  | *Hippobosca camelina* 3 | FJ265791 |  | *Dialeurodes hongkongensis* | *Dialeurodes hongkongensis* | AY264667 |  | *Triatoma melanosoma* | *Triatoma melanosoma* 2 | DQ508172 |
| *Ornithomyia avicularia* | *Ornithomyia avicularia* 1 | FJ265815 |  | *Tetraleurodes acaciae* | *Tetraleurodes acaciae* | AY264670 |  |  | *Triatoma melanosoma* 1 | DQ508188 |
|  | *Ornithomyia avicularia* 2 | FJ265814 |  | *Trialeurodes hutchingsi* | *Trialeurodes hutchingsi* | AY587140 |  | *Meccus mazzoti* | *Meccus mazzoti* 1 | DQ517448 |
|  | *Ornithomyia avicularia* 3 | FJ265811 |  | *Trialeurodes vaporariorum* | *Trialeurodes vaporariorum* | AY264672 |  |  | *Meccus mazzoti* 2 | DQ517447 |
|  | *Ornithomyia avicularia* 4 | FJ265812 |  | *Tetraleurodes mori* | *Tetraleurodes mori* | AY264671 |  | *Aphalaroida inermis* | *Aphalaroida inermis* | AF263556 |
|  | *Ornithomyia avicularia* 5 | FJ265806 |  | *Aleyrodes proletella* | *Aleyrodes proletella* | AY587141 |  | *Cuerna costalis* | *Baumannia cicadellinicola* | AY676895 |
|  | *Ornithomyia avicularia* 6 | FJ265810 |  | *Aleyrodes elevatus* | *Aleyrodes elevatus* | AY264666 |  | *Psylla floccosa* | *Psylla floccosa* | AF286128 |
|  | *Ornithomyia avicularia* 7 | FJ265809 |  | *Aleuroplatus gelatinosus* | *Aleuroplatus gelatinosus* | AY264665 |  | *Bactericera cockerelli* | *Bactericera cockerelli* | AF263557 |
|  | *Ornithomyia avicularia* 8 | FJ265807 |  | *Neomaskellia andropogonis* | *Neomaskellia andropogonis* | AY264668 |  | *Heteropsylla cubana* | *Heteropsylla cubana* | AF286126 |
|  | *Ornithomyia avicularia* 9 | FJ265808 |  | *Aleurodicus dispersus* | *Aleurodicus dispersus* | AY264664 |  | *Eulachnus pallidus* | *Eulachnus pallidus* | AY136147 |
| *Ornithomyia biloba* | *Ornithomyia biloba* 1 | FJ265816 |  | *Aleurodicus dugesii* | *Aleurodicus dugesii* 1 | AY587142 |  | *Blastopsylla occidentalis* | *Blastopsylla occidentalis* 1 | AF263558 |
|  | *Ornithomyia biloba* 2 | FJ265813 |  |  | *Aleurodicus dugesii* 2 | AF286129 |  |  | *Blastopsylla occidentalis* 2 | AF077608 |
|  | *Ornithomyia biloba* 3 | FJ265786 |  | *Acanthaleyrodes styraci* | *Acanthaleyrodes styraci* | AY264663 |  | *Cacopsylla pyri* | *Cacopsylla pyri* | AY136145 |
|  | *Ornithomyia chloropus* | FJ265787 |  | *Bemisia tabaci* | *Bemisia tabaci* 1 | AF400474 |  | *Uroleucon ambrosiae* | *Hamiltonella defensa* 1 | AF293622 |
| *Lipoptena fortisetosa* | *Lipoptena fortisetosa* 1 | FJ265797 |  |  | *Bemisia tabaci* 2 | AF400480 |  | *Aphis craccivora* | *Hamiltonella defensa* 2 | AY136136 |
|  | *Lipoptena fortisetosa* 2 | FJ265798 |  |  | *Bemisia tabaci* 3 | AF400481 |  | *Cacopsylla myrthi* | *Cacopsylla myrthi* | AF263559 |
|  | *Lipoptena fortisetosa* 3 | FJ265796 |  |  | *Bemisia tabaci* 4 | AF400478 |  | *Psylla pyricola* | *Psylla pyricola* | AF286125 |
| *Lipoptena cervi* | *Lipoptena cervi* 1 | FJ265794 |  |  | *Bemisia tabaci* 5 | AY264677 |  | *Calophya schini* | *Calophya schini* | AF263560 |
|  | *Lipoptena cervi* 2 | FJ265795 |  | *Heteropsylla texana* | *Heteropsylla texana* | AF263562 |  | *Haematopinus apri* | *Haematopinus apri* | DQ076665 |
| *Lipoptena sp.* | *Lipoptena* sp. | FJ265799 |  | *Siphoninus phillyreae* | *Siphoninus phillyreae* | AY264669 |  | *Metamasius callizona* | *Nardonella sp.* | AY126634 |
| *Melophagus ovinus* | *Melophagus ovinus* | FJ265819 |  | *Nasonia vitripennis* | *Arsenophonus nasoniae* | M90801 |  | *Trioza magnoliae* | *Trioza magnoliae* | AF077607 |
| *Nycteribia kolenati* | *Nycteribia kolenatii* 1 | FJ265804 |  | *Triatoma infestans* | *Arsenophonus triatominarum* 1 | U91786 |  | *Haematomyzus elephantis* | *Haematomyzus elephantis* | DQ076663 |
|  | *Nycteribia kolenatii* 2 | FJ265802 |  |  | *Arsenophonus triatominarum* 2 | DQ508185 |  | *Glossina morsitans centralis* | *Wigglesworthia glossinidia* | AF022878 |
| *Nycteribia sp* | *Nycteribia* sp. | FJ265803 |  | *Diaphorina citri* | *Diaphorina citri* | AB038366 |  | *Pseudolynchia canariensis* | *Pseudolynchia canariensis* | DQ115535 |
| *Penicilidia monoceros* | *Penicilidia monoceros* | FJ265817 |  | *Pseudolynchia canariensis* | *Arsenophonus insecticola* | DQ115536 |  | *Planococcus ficus* | *Planococcus ficus* | AF476108 |
|  | *Penicilidia* sp. | FJ265805 |  | *Wahlgreniella nervata* | *Wahlgreniella nervata* | AY136168 |  | *Planococcus citri* | *Planococcus citri* 1 | AF322016 |
|  | *Trichobius* sp. | FJ265818 |  | *Myzocallis sp.* | *Myzocallis* sp. 3 | AY136153 |  |  | *Planococcus citri* 2 | AF476107 |
| *Technomyrmex albipes* | *Technomyrmex albipes* 1 | FJ265789 |  | *Aphis spiraecola* | *Aphis spiraecola* | AY136142 |  | *Paracoccus nothofagicola* | *Paracoccus nothofagicola* | AF476109 |
|  | *Technomyrmex albipes* 2 | FJ265790 |  | *Beta vulgaris* | *Beta vulgaris* | AY057392 |  | *Cyphonococcus alpinus* | *Cyphonococcus alpinus* | AF476102 |
| *Aenictus huonicus* | *Aenictus huonicus* | FJ265788 |  | *Fragaria vesca* | *Phlomobacter fragariae 1* | DQ538372 |  | *Melanococcus albizziae* | *Melanococcus albizziae* | AF476106 |
| *Myzocalis sp.* | *Myzocalis* sp. 1 | FJ265801 |  |  | *Phlomobacter fragariae 2* | DQ538377 |  | *Australicoccus grevilleae* | *Australicoccus grevilleae* | AF476099 |
|  | *Myzocalis* sp. 2 | FJ265800 |  |  | *Phlomobacter fragariae 3* | DQ538374 |  | *Antonina pretiosa* | *Antonina pretiosa* | AF476101 |
| **Sequences retrieved from GenBank** | | |  |  | *Phlomobacter fragariae 4* | DQ538375 |  | *Antonina crawii* | *Antonina crawii* | AB030020 |
|  |  |  |  |  | *Phlomobacter fragariae 5* | DQ538376 |  | *Amonostherium lichtensioides* | *Amonostherium lichtensioides* | AF476100 |
| *Dermacentor variabilis* | *Dermacentor variabilis* 1 | AY265348 |  |  | *Phlomobacter fragariae 6* | DQ538377 |  | *Vryburgia amaryllidis* | *Vryburgia amaryllidis* | AF476110 |
|  | *Dermacentor variabilis* 2 | AY265347 |  |  | *Phlomobacter fragariae 7* | DQ538378 |  | *Erium globosum* | *Erium globosum* | AF476105 |
|  | *Dermacentor variabilis* 3 | AY265346 |  |  | *Phlomobacter fragariae 8* | DQ538379 |  | *Dysmicoccus neobrevipes* | *Dysmicoccus neobrevipes* | AF476104 |
|  | *Dermacentor variabilis* 4 | AY265345 |  | *Myrmeleon mobilis* | *Myrmeleon mobilis* | DQ068928 |  | *Dysmicoccus brevipes* | *Dysmicoccus brevipes* | AF476103 |
|  | *Dermacentor variabilis* 5 | AY265344 |  | *Aphis mellifera* | *Aphis mellifera* 1 | DQ837613 |  | *Anomoneura mori* | *Anomoneura mori* | AB013086 |
|  | *Dermacentor variabilis* 6 | AY265343 |  |  | *Aphis mellifera* 2 | DQ837612 |  | *Camponotus fellah* | *Blochmania fellah* | EF422835 |
|  | *Dermacentor variabilis* 7 | AY265342 |  | *Glycaspis brimblecombei* | *Glycaspis brimblecombei* 1 | EU043378 |  | *Diuraphis noxia Diuraphis noxiaDiuraphis noxia* | *Buchnera aphidicola* | M63251 |
|  | *Dermacentor variabilis* 8 | AY265341 |  |  | *Glycaspis brimblecombei* 2 | EU039464 |  | *Coptosoma parvipictum* | *Ishikawaella capsulata* | AB244769 |
| *Lipoptena cervi* | *Lipoptena cervi 3* | DQ314777 |  | *Pediculus schaeffi* | *Riesia pediculischaeffi* | EF110573 |  | *Bemisia tabaci* | *Portiera aleyrodidarum* 1 | AF400451 |
|  | *Lipoptena cervi 4* | DQ314778 |  | *Pediculus capitis* | *Riesia pediculicola* 1 | DQ076660 |  |  | *Portiera aleyrodidarum* 2 | AF400458 |
| *Trichobius longipes* | *Trichobius longipes 1* | DQ314773 |  | *Pediculus humanus* | *Riesia pediculicola* 2 | DQ076659 |  | *Macrosiphoniella ludovicianae* | *Regiella insecticola* 1 | AF293619 |
|  | *Trichobius longipes 2* | DQ314772 |  |  | *Riesia pediculicola* 3 | EF110572 |  | *Pemphigus betae* | *Regiella insecticola* 2 | AY136154 |
|  | *Trichobius longipes 3* | DQ314771 |  |  | *Riesia pediculicola* 4 | EF647590 |  | *** | *Proteus mirabilis* | AB079370 |
|  | *Trichobius longipes 4* | DQ314770 |  |  | *Riesia pediculicola* 5 | AB263104 |  | *** | *Photorhabdus luminescens* | BX571859 |
| *Trichobius parasiticus* | *Trichobius parasiticus* 1 | DQ314774 |  | *Pthirus pubis* | *Riesia pthiripubis* | EF110574 |  | *** | *Pasteurella multocida* | AE006065 |
|  | *Trichobius parasiticus* 2 | DQ314775 |  | *Triatoma rubrofasciata* | *Triatoma rubrofasciata* 1 | DQ508185 |  | *** | *Pseudomonas aeruginosa* | EU515133 |
| *Trichobius caecus* | *Trichobius caecus 1* | DQ314769 |  |  | *Triatoma rubrofasciata* 2 | DQ508169 |  | *** | *Chromatium okenii* | AJ223234 |
